# Indices of Regional Brain Atrophy: Formulae and Nomenclature

**DOI:** 10.7759/cureus.295

**Published:** 2015-08-06

**Authors:** Manuel Menendez, Oscar Arias-Carrión

**Affiliations:** 1 Neurology, Hospital Alvarez-Buylla; 2 Unidad de Trastornos del Movimiento y Sueño, Hospital General Dr. Manuel Gea Gonzalez

**Keywords:** brain atrophy, brain morphometry, mri, volumetry, planimetry, alzheimer's disease, parkinson's disease, clinical trials, neurodegenerative disease, aging

## Abstract

The pattern of brain atrophy helps to discriminate normal age-related changes from neurodegenerative diseases. Albeit indices of regional brain atrophy have proven to be a parameter useful in the early diagnosis and differential diagnosis of some neurodegenerative diseases, indices of absolute regional atrophy still have some important limitations. We propose using indices of relative atrophy for representing how the volume of a given region of interest (ROI) changes over time in comparison to changes in global brain measures over the same time.

A second problem in morphometric studies is terminology. There is a lack of systematization naming indices and the same measure can be named with different terms by different research groups or imaging softwares. This limits the understanding and discussion of studies.

In this technological report, we provide a general description on how to compute indices of absolute and relative regional brain atrophy and propose a standardized nomenclature.

## Introduction

Brain atrophies with age. Numerous cross-sectional and longitudinal imaging studies have found an inverse correlation between increasing age and decreasing brain volumes [1-7], and these findings are substantiated by postmortem data [8-9]. Grey matter (GM) volume loss appears to be a constant, linear function of age throughout adult life, whereas white matter (WM) volume loss seems to be delayed until middle adult life [10]. In any case, it is clear that brain volume decreases in healthy aging, and it can be observed in almost all brain areas over one year. Albeit changes are especially evident in temporal and prefrontal cortices, where the rate of annual decline is about 0.5%/year, all subcortical and ventricular regions, except the caudate nucleus and the fourth ventricle, show changes visible in this period of time [11].

A better understanding of the brain aging process may help to discriminate normal age-related changes from neurodegenerative diseases. In neurodegenerative diseases, besides physiologic atrophy, patients develop a progressive focal atrophy that grows in extent and intensity with time. For instance, atrophy in Alzheimer’s disease (AD) begins outside the hippocampus, with development of neurofibrillary tangles in the transentorhinal and entorhinal cortex, spreading subsequently to the subiculum and CA1 regions of the hippocampus and later on to other cortical areas [12-15]. A similar progression from focal to wide atrophy is seen in almost all neurodegenerative diseases. As a result, cortical atrophy and ventricular enlargement are present both in healthy aging and in all neurodegenerative diseases to some extent. However, the start point of regional atrophy, the rate of atrophy, and the pattern of atrophy progression varies between healthy aging and neurodegenerative disease and among neurodegenerative diseases.

Therefore, we sustain that we should use measures of relative regional atrophy comparing how a given structure changes in respect to global brain changes when studying neurodegenerative diseases. Moreover, as intracranial volume is an interindividual variant, all studies using absolute global or regional atrophy measures need to be normalized by intracranial volume. In the same line, studies found a highly significant and well-recognized effect of sex on volume, with men having larger brain volumes [2, 7]. This finding suggests that studies considering the effect of sex on cross-sectional volumes should also include a correction for head size to reduce this potential confounding effect. All these limitations are automatically solved when using relative rates of atrophy as each subject forms his or her own control.

Magnetic resonance imaging (MRI)–based measurements of the brain have been proposed as aids in the diagnosis of AD and other neurodegenerative diseases in clinical practice. Most studies assessing brain atrophy in neurodegenerative diseases have been done using volumetric techniques, although some others--specifically addressed to clinical practice--have used linear and planimetric techniques. Visual rating scales have also been developed, although these are not objective and, therefore, are not valid for assessing progression [16]. In any case, the rationale of using relative regional atrophy applies with all methodological approaches.

A second problem in morphometric studies is terminology, the lack of a homogeneous nomenclature for parameters and indices of atrophy. The same measure can be named with different names by different research groups or imaging software programs. For instance, a widely used measure, such as the yearly rate of Whole Brain Atrophy (yrWBA), is also known as yearly brain atrophy rate (yBAR) and annualised percent brain volume change (PBVC/y) [17]. This fact is a limitation for the comparison and discussion of results between groups.

The aim of this technological report is to provide a general description on how to compute indices of absolute and relative regional brain atrophy and propose a standardized nomenclature.

## Technical report

### Computing indices of absolute regional brain atrophy

Computing the yearly rate of absolute regional brain atrophy is conceptually simple. First, we need the find out the volume of the target region (named Region of Interest, ROI) in the basal and follow-up MRI and compute the difference. Then all we need to do is to “annualize” the time lapsed between the two MRI studies (12/number of months). To do this, we just need to divide the number of months transcurred from basal to follow-up MRI studies. We recommend multiplying the result by 100 to avoid working with decimals.

Thus, the general formula for computing indices of absolute rate of atrophy is as follows:

$yrA-ROI=\frac{\left ( ROI1-ROI2 \right ) \times1200}{(months\ between\ MRI\ studies)}$

where ROI is the short name of ROI; ROI1 is the volume of the ROI in the basal MRI and ROI2 the volume of the ROI in the follow-up MRI.

Indices can be computed for each hemisphere separately, or for both brain hemispheres together (taking the addition of volumes of ROI on both hemispheres). We will use cerebral hemispheres for telencephalic and diencephalic structures while we will use cerebellar hemispheres and ipsilateral brainstem for structures in the *posterior fossa*.

### Computing indices of relative regional brain atrophy

In order to compute indices of relative atrophy, we need to find out the volume of the ROI and the volume of a brain structure used as a measure of reference (here named Ref). There are several potential Ref that can be used. The Ref is usually the whole brain volume, although it can also be other parameters, such as the cortical brain volume or ventricular volume (an inverse, indirect measure of brain atrophy). In this case, we use the lateral ventricles (I-II) with telencephalic ROI and the third ventricle (III) with diencephalic structures. When the ROI is in the *posterior fossa*, the Ref can be the volume of the parenchyma (brainstem plus cerebellar volumes) or the fourth ventricle (IV) as an inverse, indirect measure.

Thus, the general formula for computing indices of yearly rates of relative regional atrophy is as follows:

$yrRA-ROI(Ref)=\frac{\left ( ROI1-ROI2 \right ) \times1200}{\left (Ref1-Ref2 \right ) \times(months\ between\ MRI\ studies)}$

where Ref is the short name of the measure of referece and ROI is the short name of the ROI.

ROI1 is the volume of the ROI in the first MRI and ROI2 the volume of the ROI in the second MRI.

Ref1 is the reference volume in the first MRI and Ref2 the reference volume in the second MRI. When using inverse measures of global atrophy such as ventricular volumes, we compute (Ref-Ref1) to avoid negative values.

Again, indices can be computed for each hemisphere separately, or for both hemispheres together. The ROI’s ipsilateral reference must always be taken with paired reference structures.

### Nomenclature

We propose a standardized, comprehensive terminology for naming the absolute and relative rates of atrophy of every ROI. The proposed nomenclature for the most frequently used ROI can be found in Table 1.

**Table 1:** Nomenclature of Regions of Interest (ROI) and related indices of regional atrophy The most frequently used ROI with the corresponding nomenclature for absolute and relative rates of atrophy. The letters "Ref" refer to the structure or paramenter used as measure of reference.

ROI	Absolute Rate of Atrophy	Relative Rate of Atrophy
Telencephalic ROI		
Cortical Gray Matter	yrA-CGM	yrRA-CGM(Ref)
Whole Brain Hemisphere	yrA-WBHp	yrRA-WBH(Ref)
Brain Hemispheric-Cortical Gray Matter	yrA-BHpCGM	yrA-BHCGM(Ref)
Lobar Cortical Gray Matter (frontal, temporal, parietal, occipital and insular lobes)	yrA-LCGM	yrA-LCGM(Ref)
Gyri (the name of any girus is suitable, following international nomina anatomica)	yrA-"Gyrus name"	yrRA-"Gyrus name"(Ref)
Forebrain Parenchyma	yrA-FP	yrRA-FP(Ref)
Hippocampus	yrA-H	yrRA-H(Ref)
Medial temporal lobe (hippocampus + parahippocamal gyrus)	yrA-MTL	yrRA-MTL(Ref)
Amygdala	yrA-Amy	yrRA-Amy(Ref)
Caudate	yrA-Cau	yrRA-Cau(Ref)
Putamen	yrA-Pu	yrRA-Pu(Ref)
Striatum (Caudate+Putamen)	yrA-St	yrRA-St(Ref)
Pallidum	yrA-Pa	yrRA-Pa(Ref)
Lenticularis nucleus (Putamen+Pallidum)	yrA-Len	yrRA-Len(Ref)
Diencephalic ROI		
Thalamus	yrA-T	yrRA-T(Ref)
Hypothalamus	yrA-HypoT	yrRA-GypoT(Ref)
Subthalamic nucleus	yrA-SubT	yrRA-SubT(Ref)
ROI in the posterior fossa		
Whole Cerebellum	yrA-WCer	yrRA-Cer(Ref)
Whole Cerebellar Hemisphere	yrA-WCerHp	yrRA-HCer(Ref)
Whole Cerebellar Cortical Gray Matter	yrA-WCerCGM	yrRA-WCerCGM(Ref)
Cerebellar Hemispheric Cortical Gray Matter	yrA-CerHpCGM	yrRA-CerHCGM(Ref)
Midbrain (Mesencephalon)	yrA-MidB	yrRA-MidB(Ref)
Pons	yrA-Pons	yrRA-Pons(Ref)
Medulla	yrA-Med	yrRA-Med(Ref)
Olivary body	yrA-OB	yrRA-OB(Ref)
Substantia Nigra	yrA-Sn	yrRA-Sn(Ref)
Red Nucleus	yrA-Rn	yrRA-Rn(Ref)

The first two letters denote the “temporal lapse”, this way all indices start with “yr” for “yearly rate”. Then we place the letter “A” if we are referring to an absolute rate of atrophy or the letters “RA” if we are referring to a relative rate of atrophy. Then a hyphen “-” separates the “yrA” or “yrRA” from the name of the ROI. Thus, the hyphen is followed by the initials of the anatomic structure used as ROI. When the ROI is a paired structure, the letter l (left) or r (right) must precede the initials of the structure if we are measuring side by side; otherwise, it will be understood that it is referred to both sides together. Finally, in rates of relative atrophy only, we add the initials of the referenced measure between parenthesis at the end “(Ref)”, where (Ref) is the short name of the parameter or structure used as measure of reference (Table 2). 

**Table 2:** Nomenclature of Structures Used as Reference Structures frequently used as reference in indices of relative rate of regional atrophy. Structures are arranged by the location of the ROI that will be used along.

Brain Structure	Initials or Symbol
To be used with Telencephalic ROI	
Whole Brain	WB
Brain Hemisphere	BHp
Cortical Gray Matter	CGM
Lateral Ventricles	I-II
To be used with Diencephalic ROI	
Whole Brain	WB
Third Ventricle	III
To be used with ROI in the posterior fossa	
Whole Brain	WB
Parenchyma in the posterior fossa	Ppf
Cerebellar Hemisphere	CerHp
Fourth Ventricle	IV

Following this nomenclature, it is easy to read any index. For instance, the “yrA-Rn” reads “*yearly rate of atrophy of the red nucleus*“ -and we understand it is a measure of absolute atrophy of both red nuclei- and the “yrRA-lT(WB)” reads “*yearly rate of relative atrophy of the left thalamus referred to whole brain*“.  

## Discussion

Regional brain atrophy has been proposed to be used in clinical practice for diagnosing neurodegenerative diseases and also as a surrogate marker of disease progression in clinical trials [16]. However, there is a lack of consensus on how to compute and nominate indices of regional brain atrophy.

Here, we describe a general approach for computing rates of absolute and relative regional atrophy--that may be extended to any ROI--at the time of proposing a standardized nomenclature. These indices are especially thought to be used with volumetric techniques, but they can also be applied to planimetric techniques where areas of ROI are used instead of volumes. Indeed, we have developed and validated some planimetric techniques, such as the yearly rate of Medial Temporal Lobe Atrophy (yrMTA) [18]--this should be named yrRA-MTL(I-II) in accordance to the terminology described here. The yrMTA has proven some usefulness in the diagnosis of AD (Poster presented at the Alzheimer’s Association International Conference, Washington, 2015) and in correlating memory deficits in Parkinson’s disease (PD) (Presented at the 18th Congress of Parkinson’s Disease and Movement Disorders, Stockholm, 2014 and the 10th International Congress on Non-Motor Dysfunctions in Motor Dysfunctions in Parkinson’s Disease, Nice, 2014). We have also described in detail some volumetric indices, such as the yearly rate of relative atrophy of the thalamic nuclei (yrRAT(I-II-III)) [19] that has proved helpful in the diagnosis of multiple sclerosis and in assessing the prognosis of patients with clinically isolated syndrome (Presented at the 31st ECTRIMS Congress, Barcelona, 2015).

If the methodological approach and nomenclature proposed here were adopted by all research groups working in brain morphometry, it would ease comparing and discussing results of studies addressing the rates of regional brain atrophy.

There is much work to do before any of these parameters are ready to be used with diagnostic purposes in routine clinical practice. Extensive research is needed in both retrospective and prospective studies. Retrospective studies are relatively easy to address, particularly for some conditions such as AD and PD, where large databases of neuroimaging studies and clinical data are available to researchers worldwide. Those indices showing positive results in retrospective studies should be validated in prospective studies before they can be used in clinical practice and clinical trials.

## Conclusions

There is a lack of consensus on how to compute and nominate indices of regional brain atrophy. Here, we provide a general description on how to compute indices of absolute and relative regional brain atrophy and propose a standardized nomenclature.

If this approach were universally adopted, it would allow a direct comparison of results from different research groups.
